# Supplementary material for: Microarray Technology May Reveal the Contribution of Allergen Exposure and Rhinovirus Infections as Possible Triggers for Acute Wheezing Attacks in Preschool Children
Source: Viruses. 2021 May 15;13(5):915. doi: 10.3390/v13050915 (PMC8155838; doi:10.3390/v13050915)
Supplement: Supplementary file 1 [file viruses-13-00915-s001.zip › viruses-1157497-SI.pdf]

Table S1. List of molecules spotted on the allergen chip.

Allergen	Source	Rec. or natural	Name/Function of the protein/Allergen family
**Respiratory allergens**			
Aln g 1	*Alder*	R	PR-10
Alt a 1	*Alternaria*	R	Acidic glycoprotein
Alt a 6	*Alternaria*	R	Enolase
Amb a 1	*Ragweed*	N	Pectate lyase
Art v 1	*Mugwort*	N	Defensin
Art v 3	*Mugwort*	N	LTP
Asp f 1	*Aspergillus*	R	Mitogillin family
Asp f 3	*Aspergillus*	R	Peroxysomal protein
Asp f 6	*Aspergillus*	R	Mn superoxide dismutase
Bet v 1	*Birch*	R	PR-10
Bet v 2	*Birch*	R	Profilin
Bet v 4	*Birch*	R	Polcalcin
Bla g 1	*Cockroach*	R	Cockroach group 1
Bla g 2	*Cockroach*	R	Aspartic protease
Bla g 5	*Cockroach*	R	Glutathione S-transferase
Bla g 7	*Cockroach*	N	Tropomyosin
Blo t 5	*Blomia tropicalis*	R	Group 5/21 mite allergens
Can f 1	*Dog*	R	Lipocalin
Can f 2	*Dog*	R	Lipocalin
Can f 3	*Dog*	N	Serum Albumin
Can f 4	*Dog*	R	Lipocalin (Odorant–binding proten)
Can f 5	*Dog*	R	Arginine esterase (Trypsin-like serine protease)
Can f 6	*Dog*	R	Lipocalin
Che a 1	*Goosefoot*	R	Trypsin Inhibitor
Cyn d 1	*Bermuda grass*	N	Grass group 1
Cry j 1	*Japanese cedar*	N	Pectate lyase
Cup a 1	*Cypress*	N	Pectate lyase
Der f 1	*House dust mite*	N	Cysteine protease
Der f 2	*House dust mite*	R	NPC2 family
Der p 1	*House dust mite*	N	Cysteine protease
Der p 2	*House dust mite*	R	NPC2 family
Der p 4	*House dust mite*	R	Alpha amylase
Der p 5	*House dust mite*	R	Group 5 mite allergens
Der p 7	*House dust mite*	R	Group 7 mite allergens

Der p 10	*House dust mite*	R	Tropomyosin
Der p 11	*House dust mite*	R	Paramyosin
Der p 14	*House dust mite*	R	Apolipophorin
Der p 15	*House dust mite*	R	Chitinase-like protein
Der p 18	*House dust mite*	R	Chitin-binding protein
Der p 21	*House dust mite*	R	Group 21 mite allergens
Der p 23	*House dust mite*	R	Peritrophin-like protein
Der p 37	*House dust mite*	R	Protein similar to chitin-binding protein
Equ c 1	*Horse*	R	Lipocalin
Equ c 3	*Horse*	N	Serum Albumin
Fag e 2	*Buckwheat*	N	Storage protein, 2S albumin
Fel d 1	*Cat*	R	Uteroglobin
Fel d 2	*Cat*	N	Serum Albumin
Fel d 4	*Cat*	R	Lipocalin
Lep d 2	*Storage mite*	R	NPC2 family
Mal d 1	*Apple*	R	PR-10
Mer a 1	*Annual mercury*	R	Profilin
Mus m 1	*Mouse*	N	Lipocalin
MUXF3	*CCD*	N	CCD
Phl p 1	*Timothy*	R	Grass group 1
Phl p 2	*Timothy*	R	Grass group 2
Phl p 4	*Timothy*	N	Berberine bridge enzyme
Phl p 5	*Timothy*	R	Grass group 5
Phl p 6	*Timothy*	R	Grass group 6
Phl p 7	*Timothy*	R	Polcalcin
Phl p 11	*Timothy*	R	Trypsin inhibitor
Phl p 12	*Timothy*	R	Profilin
Pla a 1	*Plane*	R	Invertase Inhibitor
Pla a 2	*Plane*	N	Polygalacturonase
Pla a 3	*Plane*	R	LTP
Pla l 1	*Plantain*	R	Pectate lyase
Food allergens			
Act d 1	*Kiwi*	N	Cysteine protease
Act d 2	*Kiwi*	N	Thaumatin-like protein
Act d 5	*Kiwi*	N	Kiwellin
Act d 8	*Kiwi*	R	PR-10
Ana o 1	*Cashew nut*	N	Vicilin-like protein
Ana o 2	*Cashew nut*	R	Storage protein, 11S globulin
nAna o 2	*Cashew nut*	N	Storage protein, 11S globulin

Ana o 3	*Cashew nut*	R	Storage protein, 2S albumin
Api g 1	*Celery*	R	PR-10
Ara h 1	*Peanut*	R	Storage protein, 7S globulin
Ara h 2	*Peanut*	R	Storage protein, 2S albumin
Ara h 3	*Peanut*	R	Storage protein, 11S albumin
Ara h 6	*Peanut*	N	Storage protein, 2S albumin
Ara h 8	*Peanut*	R	PR-10
Ara h 9	*Peanut*	R	LTP
Ber e 1	*Brazil nut*	R	Storage protein, 2S albumin
Bos d 4	*Cow's Milk*	N	Alpha-lactalbumin
Bos d 5	*Cow's Milk*	N	Beta-lactoglobulin
Bos d Lactoferrin	*Cow's Milk*	N	Transferrin
Bos d 8	*Cow's Milk*	N	Casein
aS1-casein	*Cow's Milk*	N	Casein
aS2-casein	*Cow's Milk*	N	Casein
b-casein	*Cow's Milk*	N	Casein
K-casein	*Cow's Milk*	N	Casein
Transferrin	*Cow's Milk*	N	Transferrin
Bovine serum albumin	*Cow's Milk*	N	Serum albumin
Bos d 6	*Cow*	N	Serum albumin
Cor a 1.0401	*Hazelnut*	R	PR-10
Cor a 8	*Hazelnut*	R	LTP
Cor a 9	*Hazelnut*	N	Storage protein, 11S globulin
Gad c 1	*Codfish*	R	Parvalbumin
Gal d 1	*Egg white*	N	Ovomucoid
Gal d 2	*Egg white*	N	Ovalbumin
Gal d 3	*Egg white*	N	Ovotransferrin
Gal d 5	*Egg yolk/chicken*	N	Livetin (serum albumin)
Gly m 4	*Soy*	R	PR-10
Gly m 5	*Soy*	N	Storage protein, Beta-conglycinin
Gly m 6	*Soy*	N	Storage protein, Glycinin
Jug r 1	*Walnut*	N	Storage protein, 2S albumin
Jug r 2	*Walnut*	N	Cupin superfamily, 7S Vicilin-like globulin
Jug r 3	*Walnut*	N	LTP
Ole e 1	*Olive*	N	Trypsin inhibitor
Ole e 7	*Olive*	N	LTP
Ole e 9	*Olive*	R	Glucanase
rOle e 5	*Olive*	R	Superoxide dismutase

rOle e 6	*Olive*	R	Oleaceae group 6
rOle e 8	*Olive*	R	Calcium-binding protein (Polcalcin)
rOle e 10	*Olive*	R	Glycosyl hydrolase
Par j 2	*Wall pellitory*	R	LTP
Pen m 1	*Shrimp*	N	Tropomyosin
Pen m 2	*Shrimp*	N	Arginine Kinase
Pen m 4	*Shrimp*	N	Calcium-binding protein
Pis v 3	*Pistachio*	R	7S Vicilin-like Globulin
Pru p 1	*Peach*	R	PR-10
Pru p 3	*Peach*	R	LTP
Pru du 3	*Almond*	R	LTP
Pru du 4	*Almond*	R	Profilin
Pru du 6	*Almond*	N	Storage protein, 11S globulin
Pru du 6.01	*Almond*	R	Storage protein, 11S globulin
Pru du 6.02	*Almond*	R	Storage protein, 11S globulin
Sal k 1	*Saltwort*	N	Pectin methylesterase
Ses i 1	*Sesame*	N	Storage protein, 2S albumin
Tri a 14	*Wheat*	R	LTP
Tri a 19.0101	*Wheat*	N	Omega 5 gliadin
Tri a aA_TI	*Wheat*	N	Alpha-Amylase / Trypsin Inhibitor
Tri a 36 191	*Wheat*	R	Fragment of LMW Glutenin
Tri a 36	*Wheat*	R	Glutenin Subunit, LMW Glutenin
GG1	*Wheat*	R	Gamma gliadin 1
Peptide 4	*Wheat*	R (peptide)	Peptide from gamma gliadin 1
Clone 79	*Wheat*	R	Gamma gliadin
Clone 85	*Wheat*	R	Gamma gliadin
Clone 110	*Wheat*	R	Gamma gliadin (Fragment)
Avenin	*Wheat*	R	Avenin
m43	*Wheat*	R	Fragment of HMW Glutenin Bx7
m82	*Wheat*	R	Fragment of HMW Glutenin Bx7
#10/Serine proteinase inhibitor	*Wheat*	R	Serine proteinase inhibitor
#37/Thioredoxin	*Wheat*	R	Thionin
#38/Glutathione transferase	*Wheat*	R	Glutathione transferase
#112/1 Cys-peroxiredoxin	*Wheat*	R	Peroxiredoxin (1-Cys-peroxiredoxin)
#123/Profilin	*Wheat*	R	Profilin
#126/Dehydrin	*Wheat*	R	Dehydrin
Alpha	*Wheat*	R	Alpha-purothionin

purothionin			
LTP	*Wheat*	R	LTP
Other allergens			
Ani s 1	*Anisakis*	R	Serine protease inhibitor
Ani s 3	*Anisakis*	R	Tropomyosin
Api m 1	*Bee venom*	R	Phospholipase A2
Api m 2	*Bee venom*	R	Hyaluronidase
Api m 4	*Bee venom*	R	Melittin
Hev b 1	*Latex*	R	Rubber elongation factor
Hev b 3	*Latex*	R	Small rubber particle protein
Hev b 5	*Latex*	R	Acidic protein
Hev b 6.01	*Latex*	R	Hevein
Hev b 8	*Latex*	R	Profilin
Ves v 1	*Wasp*	R	Phospholipase
Ves v 5	*Wasp*	R	Antigen 5
htTG	*Human*	R	Human tissue transgluthamine
